# 1776. Coinfection of severe fever with thrombocytopenia syndrome virus and *Coxiella burnetii* infection by the developmental stage of hard ticks in the subtropical region of South Korea

**DOI:** 10.1093/ofid/ofad500.1605

**Published:** 2023-11-27

**Authors:** Yuyeon Kim, Sang Taek Heo, Jeong Rae Yoo, Misun Kim, Keun Hwa Lee

**Affiliations:** Jeju National University Hospital, Jeju-si, Cheju-do, Republic of Korea; Department of Infectious Disease, Jeju National University School of Medicine, Jeju, South Korea, Jeju, Cheju-do, Republic of Korea; Jeju National University, College of Medicine, Jeju, Cheju-do, Republic of Korea; Jeju National University Hospital, Jeju-si, Cheju-do, Republic of Korea; Hanyang University College of Medicine, Jeju, Cheju-do, Republic of Korea

## Abstract

**Background:**

Severe fever with thrombocytopenia syndrome virus (SFTSV) is transmitted through tick bites. Ticks are potential vectors for the bacterium *Coxiella burnetii* (*C. burnetii*) that causes Query fever. Here, we analyzed SFTSV and *C. burnetii* co-infection rates in ticks in rural areas of Jeju Island, South Korea.

**Methods:**

Free ticks were collected from the natural environment of the island between 2016 and 2019, and SFTSV RNA was extracted. Additionally, ribosomal RNA gene sequencing was used to identify *Coxiella* species.

**Results:**

*Haemaphysalis longicornis* (*H. longicornis*) was the most common tick species followed by *H. flava*. Tick number gradually increased from April, peaked in August, and was lowest in March. Of all the collected ticks, 82.6% (2,851/3,458) were nymphs, 17.9% (639/3,458) adults, and 0.1% (4/3,458) larvae. SFTSV-infected ticks comprised 12.6% of all ticks; their numbers were the lowest in November–December, increased from January, and were mostly identified in the adult stage during June–August. *C. burnetii* infections were detected in 4.4% of the SFTSV-infected *H. longicornis* ticks. *Coxiella burnetii* co-infection was mainly observed in the nymph stage of *H. longicornis*, with the highest infection rate in January, followed by December and November.

**Conclusion:**

Our findings suggest that Jeju Island has a high SFTSV and potential *C. burnetii* infection in ticks. This study provides important insights regarding SFTS and Q fever risk to humans in South Korea.

**Disclosures:**

**All Authors**: No reported disclosures

